# Spatial and Environmental Drivers of Macroinvertebrate Diversity in Groundwater‐Dependent Pan Wetlands Across a Transboundary Aquifer System

**DOI:** 10.1002/ece3.74440

**Published:** 2026-10-19

**Authors:** Busisiwe P. Gumede, Linton F. Munyai, Naicheng Wu, Collins Oduro, Tatenda Dalu

**Affiliations:** ^1^ School of Biology and Environmental Sciences University of Mpumalanga Nelspruit South Africa; ^2^ Aquatic Systems Research Group, Department of Water and Sanitation University of Limpopo Polokwane South Africa; ^3^ School of Geography and Remote Sensing, Zhejiang‐Germany Joint Laboratory on Remote Sensing of Coastal Ecosystem Ningbo University Ningbo China; ^4^ Centre for Invasion Biology, School for Climate Studies Stellenbosch University Stellenbosch South Africa

**Keywords:** environmental variables, groundwater‐dependent wetlands, hydroperiod dynamics, macroinvertebrate diversity, spatial connectivity

## Abstract

Groundwater‐dependent pan wetlands are keystone habitats for aquatic biodiversity in semi‐arid landscapes, yet the processes that structure their invertebrate communities remain poorly resolved. We surveyed 221 pan wetlands across the Khakhea–Bray Transboundary Aquifer (KBTA), South Africa, a landscape of low relief (1027–1231 m a.s.l.), and asked whether macroinvertebrate assemblages track local physicochemical conditions, landscape position, or both. Pans were assigned to four elevation classes, used here as a surrogate for hydrogeomorphic setting and its hydroperiod consequences rather than as a climatic elevation gradient, because the total relief is an order of magnitude smaller than in studies where elevation acts as a direct driver. Waters were predominantly neutral to alkaline (mean pH 7.6–8.6), consistent with a strong groundwater signature. Dissolved oxygen, water temperature, water depth, ammonium, nitrate and phosphate differed significantly among classes, whereas pH, oxidation–reduction potential, conductivity, total dissolved solids and surface area did not. Assemblage composition differed among classes, but the effect was weak: global ANOSIM detected no separation, and similarity percentage analysis attributed between‐class dissimilarity (67.9%–73.4%) to shifts in the relative abundance of a few dominant taxa, chiefly Anostraca, Spinicaudata and Notostraca, rather than to species turnover. Taxa richness differed significantly among classes, whereas abundance, Simpson, Shannon–Wiener and evenness did not, and within‐class variation exceeded among‐class differences. Shallow, short‐hydroperiod pans were dominated by large branchiopods, whereas deeper, longer‐hydroperiod pans supported proportionally more predatory aquatic insects and molluscs, consistent with hydroperiod and predation, rather than elevation, acting as the operative filters. Boosted regression trees ranked geographic position, particularly longitude, above local physicochemical variables as a correlate of taxa richness; because the surveyed pans are strongly clustered in space, this result is interpreted as evidence of unmeasured, spatially structured habitat variation rather than as a predictive relationship. Conserving these systems will require maintaining groundwater regimes and the full range of hydroperiods across the pan network under coordinated transboundary management, and monitoring assemblage composition rather than richness alone.

## Graphical abstract

This study provides one of the first integrative assessments of macroinvertebrate metacommunity structure in groundwater‐dependent pan wetlands within a transboundary aquifer system, explicitly disentangling the relative roles of environmental filtering and spatial processes. By demonstrating that biodiversity patterns are governed more strongly by landscape‐scale spatial gradients than by local water chemistry, the work challenges conventional site‐based approaches to wetland ecology and monitoring. The identification of mid‐elevation pans as biodiversity hotspots and the dominance of abundance‐driven compositional shifts rather than species loss offer new insights into resilience mechanisms in semi‐arid freshwater systems.

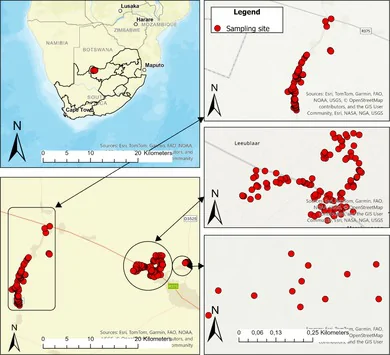

## 1 Introduction

Wetlands lie at the interface between terrestrial and aquatic systems and deliver disproportionate biodiversity and ecosystem‐service value relative to their extent, yet they are among the most rapidly disappearing ecosystems globally, with roughly half of the global resource lost or severely altered and comparable losses reported nationally in South Africa (Yousaf et al. 2021; Adeeyo et al. 2022). In semi‐arid regions this loss is compounded by dependence on groundwater: where abstraction, land‐use change and climate variability lower water tables, inflows decline and hydroperiods shorten, with immediate consequences for the biota (Nkosi et al. 2021; Chiloane et al. 2022). Endorheic pan wetlands, which dominate the wetland resource of the semi‐arid interior of southern Africa, are especially exposed because they are small, shallow, hydrologically isolated for much of the year and largely absent from formal monitoring networks (Rebelo et al. 2018; Rampheri et al. 2023).

Aquatic macroinvertebrates provide the most tractable biological signal of condition in these systems: they respond rapidly to water chemistry, temperature, oxygen availability and habitat structure, and they mediate decomposition and energy transfer to vertebrate consumers (Dalu et al. 2017; Allan et al. 2021; Mureithi et al. 2025). In temporary waters, hydroperiod is repeatedly identified as the master variable. Short‐hydroperiod systems are dominated by large branchiopods and other taxa with rapid development and desiccation‐resistant propagules, whereas longer‐hydroperiod systems accumulate aquatic insects and molluscs that require longer development and that also act as predators on early colonists (Van den Broeck et al. 2015; Batzer and Boix 2016; Shrestha et al. 2021). Depth, salinity and nutrient status modify this template by governing oxygen availability, resource supply and physiological tolerance limits (Oertli and Parris 2019; Brysiewicz et al. 2022). Studies of southern African pans report the same broad pattern, but are concentrated in the wetter east and north of the subcontinent and rarely encompass more than a handful of systems (Dube et al. 2019; Masina et al. 2023).

Local environmental filtering, however, explains only part of the variation in assemblages distributed across discrete habitat patches. Pan networks are textbook metacommunities, in which species sorting operates alongside dispersal limitation and mass effects, and in which the relative contribution of each depends on inter‐patch distance, dispersal mode and the structure of the intervening landscape (Heino et al. 2017; Sarremejane et al. 2022; Guerrero et al. 2025). Where patches are numerous and closely spaced, dispersal can homogenise assemblages and mask environmental signals; where they are sparse or separated by an inhospitable matrix, spatial structure can dominate. Distinguishing these possibilities requires surveys that span many pans within a single hydrogeological setting, and such surveys remain rare in semi‐arid Africa.

The Khakhea–Bray Transboundary Aquifer (KBTA), shared by Botswana and South Africa, contains hundreds of groundwater‐dependent pans and is under increasing abstraction pressure, yet its aquatic invertebrate fauna has been described for only a small subset of systems and no study has evaluated environmental and spatial structuring across the pan network as a whole (Dube et al. 2019; Masina et al. 2023; Gomo and Ngobe 2024). This gap limits the ecological basis for transboundary groundwater management, which is presently informed almost entirely by hydrogeological rather than ecological data. We therefore surveyed 221 pans across the KBTA and addressed three questions: (i) how do physicochemical conditions and macroinvertebrate assemblages vary among pans occupying different positions in this low‐relief landscape; (ii) which local environmental variables are associated with assemblage structure and taxa richness; and (iii) does geographic position account for variation in richness beyond that explained by local conditions? We hypothesised that assemblage composition would differ among pans primarily in relation to hydroperiod‐related variables, namely water depth, dissolved oxygen and nutrient concentrations, rather than with elevation itself, given the limited relief of the study area; that taxa richness and diversity would vary more within than among elevation classes; and that geographic position would account for additional variation in richness, reflecting landscape‐scale habitat structure not captured by point measurements of water chemistry.

## 2 Materials and Methods

### 2.1 Study Area

The Khakhea–Bray Transboundary Aquifer is a semi–arid groundwater system spanning Botswana and South Africa, covering approximately 29,700 km^2^ and centred around the ephemeral Molopo River (Figure 1; Gomo and Ngobe 2024). With low population density (1–10 people km^−2^), the aquifer is a critical water resource that sustains both human needs and groundwater–dependent ecosystems, including seasonally inundated wetlands and pan systems that experience pronounced hydrological variability. The region is characterised by low and uneven annual rainfall (376 mm), concentrated between November and March, coupled with high evapotranspiration rates that intensify water limitation. Temperatures range from 22°C to 34°C in summer to 2°C–20°C in winter (DWAF 2004), driving strong seasonal fluctuations in water availability. Vegetation reflects these arid conditions, with sparse thorn trees and patchy grass cover dominating sandy substrates, while denser vegetation occurs along river channels where water availability is higher (Mpakairi 2022). Topographically, the KBTA is largely flat (1027–1231 m a.s.l.), with low–lying sandy depressions and river channels facilitating the formation of temporary wetlands and pans. These habitats are ecologically important, supporting diverse aquatic communities, particularly macroinvertebrates, which depend on them for survival in an otherwise water–limited landscape. Total relief across the study area is approximately 200 m, which is small relative to the elevation ranges over which climatic zonation of aquatic communities is normally detectable; within this landscape, elevation covaries with topographic position, pan morphometry and depth to the water table rather than with climate.

**FIGURE 1:**
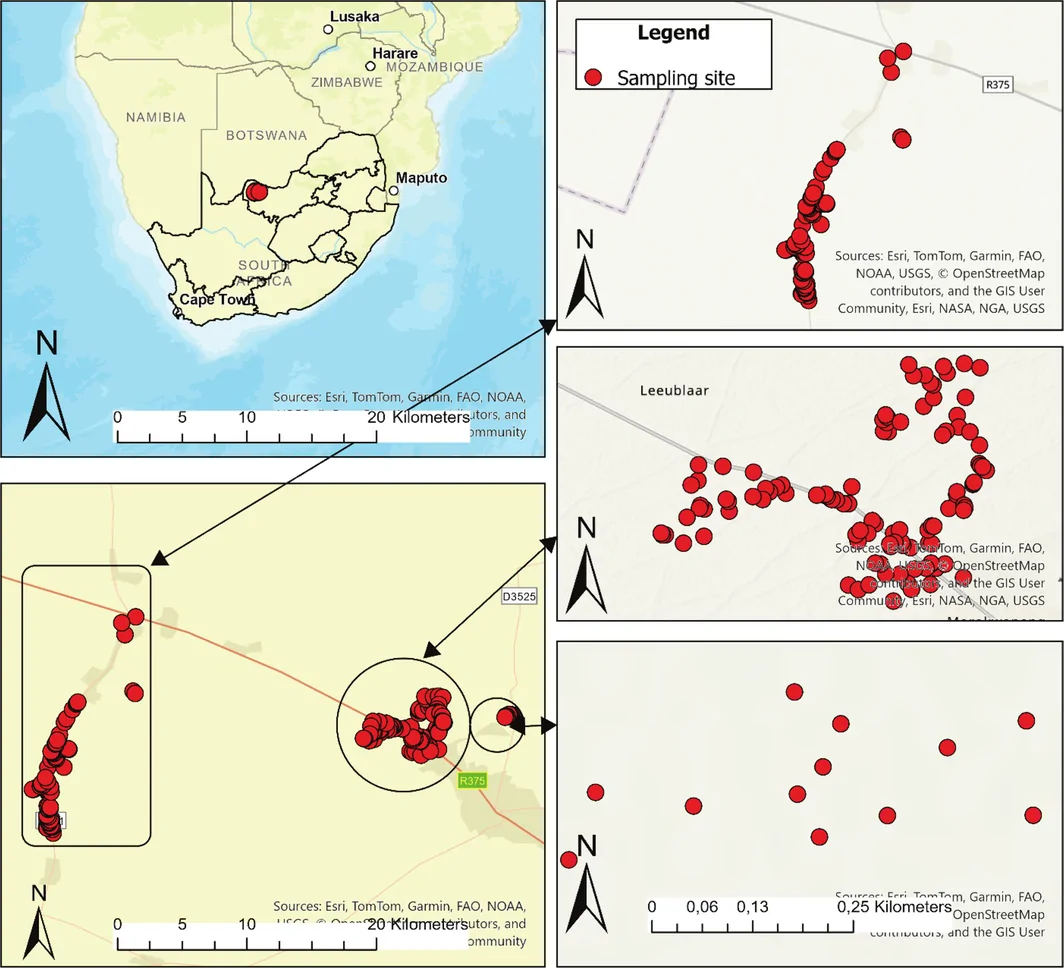
Location of the sampled pan wetlands in the Morokweng area of the Khakhea–Bray Transboundary Aquifer, North West Province, South Africa. The inset panels show the three pan clusters surveyed; each red symbol denotes one of the 221 sampled pans.

A total of 221 pan wetlands was sampled within the KBTA region (Figure 1). Because the aquifer spans only about 200 m of relief (1027–1231 m a.s.l.), elevation was not treated as a climatic gradient. Pans were instead assigned to four elevation classes: < 1100 m (*n* = 55), 1100–1149 m (*n* = 40), 1150–1199 m (*n* = 123) and > 1200 m (*n* = 6), that reflect the distribution of pan elevations within the aquifer and that correspond, in this landscape, to differences in topographic position, pan morphometry and depth to the water table. These classes are therefore used as a surrogate for hydrogeomorphic setting and its hydroperiod consequences, and not as an elevation gradient in the sense used in montane studies. The uneven representation of the classes, in particular the three pans above 1200 m, follows from the elevation distribution of pans within the aquifer and is taken into account throughout the interpretation of among‐class comparisons. For each pan, surface area and maximum water depth were recorded to characterise habitat structure and hydroperiod‐related variation.

### 2.2 Data Collection

Wetland pans across the KBTA were surveyed using standardised field and laboratory protocols to quantify environmental conditions and macroinvertebrate assemblages. Surface area was determined through GPS‐based perimeter mapping, whereby each pan boundary was walked and spatial coordinates recorded, with final area calculated using polygon area calculator in ArcGIS Pro. Water depth was measured in situ using a wading approach, recording the vertical distance from the water surface to the substrate at multiple points to capture within–pan variability.

Physicochemical variables were measured in situ at each of the 221 pans using a calibrated portable multiparameter probe (Hanna HI98192): electrical conductivity (μS cm^−1^), resistivity (MΩ cm), salinity, pH, water temperature (°C), dissolved oxygen (mg L^−1^), oxidation–reduction potential (mV) and total dissolved solids (mg L^−1^). Salinity is not analysed further because it is derived by the probe from, and is therefore collinear with, electrical conductivity. Water samples (250 mL) were collected in pre‐rinsed polyethylene bottles, stored on ice and transported to the laboratory, where phosphate (PO₄^3−^), ammonium (NH₄^+^–N) and nitrate (NO₃^−^–N) concentrations (mg L^−1^) were determined using a HI 83203 multiparameter photometer following standard colourimetric procedures, with duplicate measurements to assess analytical precision.

Macroinvertebrates were collected with a standard D‐frame dip net (30 × 30 cm frame, 1 mm mesh) swept along 10–15 m transects in each pan. Sampling combined systematic sweeping of the water column with active disturbance of the substrate and of submerged and emergent vegetation, followed by rinsing to remove detritus while retaining specimens. Samples were preserved in 70% ethanol and returned to the laboratory, where macroinvertebrates were sorted and identified under a stereomicroscope to the lowest practicable taxonomic level, genus for most Coleoptera, Hemiptera, Odonata and Mollusca, and family or subfamily for groups whose regional taxonomy does not permit finer resolution, using standard southern African identification guides and keys (Gerber and Gabriel 2002; de Moor et al. 2003). Taxa reported at family or subfamily level in the tables therefore reflect the limit of reliable identification rather than a difference in sampling unit.

### 2.3 Data Analysis

Environmental variables were tested for normality using the Shapiro–Wilk test and for homogeneity of variances using Levene's test. Variables that did not meet model assumptions were log‐transformed prior to analysis. One‐way analysis of variance (ANOVA) was used to test for differences in environmental variables and macroinvertebrate diversity metrics among the four elevation bands (< 1100 m, 1100–1149 m, 1150–1199 m, and > 1200 m). Where significant differences were detected, Tukey's Honestly Significant Difference (HSD) test was used for pairwise comparisons. Because the number of pans per elevation class was strongly unequal (*n* = 55, 40, 123 and 6), and the highest class contained only three pans, all comparisons involving the > 1200 m class are interpreted as indicative rather than confirmatory.

Differences in macroinvertebrate community composition among elevation bands were assessed using multivariate analyses. Species abundance data were square‐root transformed to reduce the influence of highly abundant taxa, and Bray–Curtis dissimilarities were calculated from the site‐by‐taxon matrix. Permutational multivariate analysis of variance (PERMANOVA) was used to test for differences in assemblage composition among elevation groups, using 9999 permutations. Where significant overall differences were detected, pairwise PERMANOVA comparisons were conducted with Bonferroni‐adjusted *p*‐values. Given the unbalanced design, PERMANOVA outcomes were interpreted alongside the rank‐based tests below, since unbalanced designs can return significant test statistics when within‐group multivariate dispersion differs among groups even in the absence of differences in assemblage location.

Analysis of similarities (ANOSIM) was used as a complementary rank‐based test of assemblage separation among elevation classes, and the global and pairwise *R* statistics were used as effect‐size measures rather than relying on *p*‐values alone. Similarity percentage analysis (SIMPER) was used to identify the taxa contributing most to within‐class similarity and between‐class dissimilarity. Relationships between macroinvertebrate diversity metrics and spatial variables (altitude, latitude, longitude) were examined using Pearson correlation on the site‐level data; because these were exploratory and numerous, *p*‐values were not adjusted for multiple comparisons and correlations are interpreted in terms of the magnitude of *r*. Statistical significance was assessed at *p* < 0.05.

Boosted regression trees (BRT) were used to rank the relative influence of spatial and local environmental predictors on macroinvertebrate taxa richness. Models were fitted in R (R Core Team 2025) using the gbm package, following Elith et al. (2008), with a Gaussian error distribution. Candidate predictors were longitude, latitude and altitude together with pH, dissolved oxygen, oxidation–reduction potential, electrical conductivity, total dissolved solids, water temperature, water depth, surface area, ammonium, nitrate and phosphate. Because the surveyed pans are strongly clustered in space rather than randomly distributed (Figure 1), predictive performance was assessed using spatially blocked cross‐validation, in which pans belonging to the same cluster were withheld together across spatial folds (Roberts et al. 2017). Under random cross‐validation the model returned a cross‐validated correlation of 0.99. The difference between these values indicates the extent to which apparent predictive accuracy reflects spatial autocorrelation among neighbouring pans rather than transferable ecological relationships, and BRT outputs are accordingly interpreted as a description of variance partitioning within the surveyed pans rather than as a predictive model.

## 3 Results

### 3.1 Environmental Variables

Across the KBTA, pan waters were predominantly neutral to alkaline (class mean pH 7.6–8.6; Table S1). The widest pH below 1100 m and at 1100–1149 m (both 3.6–10.1), whereas the three pans above 1200 m ranged from 7.9 to 8.9; differences among classes were not significant (*F* = 1.987, *p* = 0.115; Table 1). Mean oxidation–reduction potential ranged from 513.7 ± 732.6 mV below 1100 m to 97.7 ± 75.8 mV at 1150–1199 m and 102.1 ± 99.7 mV above 1200 m, with negative values (minimum −98.7 mV) recorded only in the highest class, but again did not differ significantly among classes (*F* = 0.515, *p* = 0.672; Table 1). Dissolved oxygen did differ significantly (*F* = 12.372, *p* < 0.001; Table 1), with class means of 5.2 ± 0.3, 5.1 ± 0.1 and 4.7 ± 1.8 mg L^−1^ in the three lower classes and 2.4 ± 0.4 mg L^−1^ above 1200 m (Table S1).

**TABLE 1:** Analysis of variance of environmental variables and macroinvertebrate metrics recorded among pan wetlands at different elevations within the Khakhea–Bray Transboundary Aquifer region, South Africa.

Variable	df	*F*	*p*
*Environmental*			
pH	3	1.987	0.115
Oxygen reduction potential	3	0.515	0.672
Dissolved oxygen	**3**	**12.372**	**< 0.001**
Conductivity	3	0.207	0.891
Resistivity	3	1.158	0.326
Total dissolved solids	3	1.380	0.248
Temperature	**3**	**20.735**	**< 0.001**
Surface area	3	0.474	0.700
Water depth	**3**	**16.345**	**< 0.001**
Ammonium	**3**	**10.581**	**< 0.001**
Nitrate	**3**	**18.923**	**< 0.001**
Phosphate	**3**	**10.998**	**< 0.001**
*Macroinvertebrate metrices*			
Taxa richness	**3**	**5.045**	**0.002**
Abundance	3	1.007	0.390
Simpson	3	0.195	0.900
Shannon–Wiener	3	0.829	0.478
Evenness	3	0.661	0.577

*Note:* Bold indicate significant differences at *p* < 0.05.

Electrical conductivity and total dissolved solids were highest at 1150–1199 m (301 ± 99.9 μS cm^−1^ and 157.8 ± 34.5 mg L^−1^) and lowest above 1200 m (167.6 ± 37.4 μS cm^−1^ and 86.1 ± 16.1 mg L^−1^), but neither differed significantly among classes (*F* = 0.207, *p* = 0.891 and *F* = 1.380, *p* = 0.248; Table 1). Water temperature differed significantly among classes (*F* = 20.735, *p* < 0.001) without a monotonic trend across the elevation sequence, ranging from 13.0°C ± 0.5°C below 1100 m to 17.7°C ± 1.1°C above 1200 m (Table S1). Surface area spanned three orders of magnitude (50–30,000 m^2^), was greatest at 1100–1149 m (10 033.3 ± 15466.1 m^2^) and did not differ significantly among classes (*F* = 0.474, *p* = 0.700). Water depth differed significantly (*F* = 16.345, *p* < 0.001) and was likewise non‐monotonic, with the shallowest pans at 1150–1199 m (8.3 ± 4 cm) and the deepest above 1200 m (34.7 ± 26.6 cm; Table S1). Ammonium, nitrate and phosphate all differed significantly among classes (*F* = 10.581, 18.923 and 10.998, respectively; all *p* < 0.001; Table 1): ammonium was highest below 1100 m (21.1 ± 32.4 mg L^−1^) and lowest at 1150–1199 m (1.6 ± 2.3 mg L^−1^), nitrate peaked at 1100–1149 m (71.7 ± 41.8 mg L^−1^) and phosphate at 1150–1199 m (6.5 ± 1.9 mg L^−1^; Table S1). Pairwise contrasts are given in Table S2.

### 3.2 Macroinvertebrates

PERMANOVA indicated significant differences in assemblage composition among elevation classes (Pseudo‐*F* (MC) = 1.984, *p* = 0.0001), with significant pairwise contrasts (*p* < 0.05) between < 1100 and 1100–1149 m, < 1100 and 1150–1199 m, and 1100–1149 and 1150–1199 m. Because class sizes were strongly unequal (*n* = 55, 40, 123 and 6), these contrasts are interpreted alongside the effect sizes reported below. Assemblages were numerically dominated by few taxa in all classes (Table 2). Large branchiopods accounted for the greatest share of relative abundance throughout, with Anostraca ranging from 24.8% ± 21.5% at 1100–1149 m to 29.8% ± 21.8% below 1100 m. Spinicaudata and Notostraca were most abundant below 1100 m (12.2% ± 8.8% and 10.7% ± 7.4%), Notostraca declined to 1.8% ± 2% at 1150–1199 m, and Spinicaudata was not recorded above 1200 m (Table 2).

**TABLE 2:** The relative abundances (%) of macroinvertebrate taxa recorded among pan wetlands at different elevations within the Khakhea–Bray Transboundary Aquifer region, South Africa. Taxa are listed at the lowest level to which they could be reliably identified; blank cells indicate that a taxon was not recorded in that elevation class.

Taxa	< 1100 m		1100–1149 m		1150–1199 m		> 1200 m	
	Range	Mean ± SD	Range	Mean ± SD	Range	Mean ± SD	Range	Mean ± SD
Anostraca	0–92	29.8 ± 21.8	0–86.2	24.8 ± 21.5	0–89.6	27.3 ± 21.8	4–52.8	26.7 ± 21
Spinicaudata	0–25.4	12.2 ± 8.8	0–11.3	4.3 ± 5.5	0–23.6	10.9 ± 9.6		
Notostraca	3.9–25	10.7 ± 7.4	0–4.4	2.2 ± 1.8	0–4.3	1.8 ± 2	0–20.6	8.7 ± 8
*Rhantus* sp.	0–8	3 ± 3.3	0–9.5	1.8 ± 3.7	0–2.8	0.8 ± 1.3	0–16.3	3.5 ± 6.5
*Hydaticus exclamationis*	0–2.6	0.4 ± 1	0–2.8	0.4 ± 1.1	0–4.6	0.7 ± 1.8	0–2.8	0.4 ± 1.1
Daphniidae	0–4	0.8 ± 1.6	0–2.8	0.7 ± 1.1	0–4.6	1.2 ± 1.8	0–5.7	0.9 ± 2.3
*Laccobius* sp.	0–5.3	1.2 ± 2	0–1.4	0.5 ± 0.6	0–2.9	0.4 ± 1.2	0–4.7	1.2 ± 2
Acidocerinae	0–4	1 ± 1.5	0–2	0.3 ± 0.8	0–2.8	0.4 ± 1.1	0–8.3	1.3 ± 3.4
Gyrinidae	0–17.1	2.8 ± 6.9	0–7.8	1.3 ± 3.1	0–5.8	1.9 ± 2	0–4	0.6 ± 1.6
Hydroporinae	0–4.2	1.2 ± 1.6	0–2.8	0.4 ± 1.1			0–4.7	0.7 ± 1.9
*Stenelmis* sp.	0–1.3	0.2 ± 0.5	0–1.3	0.4 ± 0.6			0–1.1	0.3 ± 0.5
Elmidae	0–3.9	0.6 ± 1.6			0–5.7	1.6 ± 2.4	0–2.8	0.8 ± 1.2
*Cybister* sp.	0–10.5	3.4 ± 4.1	0–11.2	2.3 ± 4.4	0–2.9	0.9 ± 1.2		
*Hydrocanthus* sp.	0–6.7	1.4 ± 2.7	0–5.2	2.1 ± 2.2	0–1.1	0.1 ± 0.4	0–5.4	2.1 ± 2.5
Dytiscidae	0–7.8	1.6 ± 3			0–8.8	1.9 ± 3.5	0–2	0.3 ± 0.8
*Haliplus* sp.	0–4.3	0.9 ± 1.7	0–2	0.5 ± 0.9	0–2.1	0.5 ± 0.9	0–2.2	0.3 ± 0.9
*Anisops* sp.	0–1.3	0.2 ± 0.5	0–2.6	0.4 ± 1	0–4.6	1.2 ± 1.8	0–6.1	1.2 ± 2.4
*Appasus* sp.	0–6.7	1.6 ± 2.8	0–2	0.3 ± 0.8	0–8.8	1.4 ± 3.6	0–2.1	0.3 ± 0.8
Corixidae	0–2.7	0.4 ± 1.1	0–7.8	2.1 ± 3	0–6	1 ± 2.4	0–10	1.6 ± 4
*Gerris swakopensis*	0–1.3	0.2 ± 0.5	0–7.1	2.1 ± 3.1	0–2.1	0.6 ± 0.9	0–6.1	2.9 ± 2.6
Hydrometridae	0–10.6	3.5 ± 4.1			0–3.8	1.1 ± 1.7	0–6.8	1.1 ± 2.8
*Laccocoris* sp.					0–22.8	5.6 ± 9.1	0–10	1.6 ± 4
*Paraplea* sp.	0–3.2	0.5 ± 1.3	0–6.1	1.2 ± 2.4	0–0.4	0 ± 0.1	0–2.8	0.4 ± 1.1
Notonectidae	0–4.1	0.6 ± 1.7	0–1.4	0.2 ± 0.5				
Nepidae	0–1.3	0.2 ± 0.5	0–0.4	0 ± 0.1	0–8.5	2.8 ± 3.1	0–1	0.1 ± 0.4
*Sigara* sp.	0–1.3	0.2 ± 0.5	0–2.3	0.3 ± 0.9	0–0.4	0 ± 0.1	0–6.1	1.8 ± 2.4
Veliidae	0–1	0.1 ± 0.4	0–5.1	1 ± 2	0–1.1	0.1 ± 0.4		
*Micronecta* sp.			0–2.8	0.6 ± 1.1	0–4.3	0.8 ± 1.7	0–10	1.6 ± 4
*Hydrocanthus* sp.	0–5.9	2.1 ± 2.4	0–7.8	1.3 ± 3.2	0–5.8	0.9 ± 2.4	0–8.1	3 ± 3.7
*Mesovelia vittigera*					0–1	0.1 ± 0.4	0–3.3	0.9 ± 1.4
*Ranatra* sp.					0–1.9	0.4 ± 0.8	0–0	0 ± 0
*Sympetrum* sp.	0–3.9	1.1 ± 1.5	0–8.3	2.5 ± 3.5	0–5.8	0.9 ± 2.4	0–2.3	0.3 ± 0.9
Chironomidae							0–3.3	0.5 ± 1.3
Ceratopogonidae	0–3.9	1 ± 1.7	0–10	1.6 ± 4	0–5.7	0.9 ± 2.3	0–4.7	0.7 ± 1.9
Culicidae	0–2.6	0.6 ± 1	0–7.1	1.4 ± 2.8	0–1.6	0.2 ± 0.6	0–12.2	2 ± 4.9
*Dixella* sp.			0–1.4	0.2 ± 0.6	0–3.8	1.3 ± 1.6		
Tabanidae	0–3.9	0.9 ± 1.6	0–18.4	3 ± 7.5	0–11.7	2.4 ± 4.7		
*Lestes* sp.	0–3.9	0.9 ± 1.6	0–0.5	0 ± 0.2			0–1.6	0.4 ± 0.7
*Anax* sp.	0–4.1	1.3 ± 1.7	0–7.8	3 ± 3.6	0–11.7	2.6 ± 4.5	0–8.5	1.4 ± 3.4
*Crocothemis erythraea*	0–2.1	0.5 ± 0.9	0–4.2	0.8 ± 1.6	0–2.8	0.4 ± 1.1		
*Diplacodes lefebvri*	0–3.9	0.6 ± 1.6	0–3	0.7 ± 1.2				
*Orthetrum chrysostigma*	0–2.5	0.6 ± 1			0–3.8	1.1 ± 1.7	0–4.7	1.4 ± 1.8
*Trithemis arteriosa*			0–21.4	5.5 ± 8.8	0–1.1	0.3 ± 0.5	0–1.1	0.1 ± 0.4
*Pseudagrion* sp.	0–2.7	1.1 ± 1.3	0–3.9	0.6 ± 1.5	0–5.8	0.9 ± 2.4	0–2.8	0.6 ± 1.1
*Phaon iridipennis*	0–1.6	0.5 ± 0.7	0–14.2	2.5 ± 5.7	0–1	0.1 ± 0.4		
*Ceratophallus natalensis*	0–2.7	1.1 ± 1.3	0–2.6	0.4 ± 1	0–5.7	1.5 ± 2	0–11.4	2 ± 4.5
*Corbicula fluminalis africana*	0–1.6	0.2 ± 0.6	0–0.6	0.1 ± 0.2			0–3.3	0.5 ± 1.3
Gastropoda	0–1.3	0.4 ± 0.6	0–15.3	3.8 ± 5.8	0–4.6	1.7 ± 1.9	0–4.3	0.7 ± 1.7
*Gyraulus connollyi*	0–4.1	1.1 ± 1.6			0–9.7	2.1 ± 3.9	0–8.5	3 ± 3.5
*Pisidium langleyanum*			0–1.2	0.3 ± 0.5	0–2.9	0.5 ± 1.1	0–2	0.3 ± 0.8
*Lamellidens* sp.	0–1.3	0.2 ± 0.5	0–5.7	1.3 ± 2.1	0–5.7	1.1 ± 2.2	0–2.8	1 ± 1.2
*Lymnaea truncatula*	0–1.3	0.4 ± 0.6	0–0.6	0.1 ± 0.2	0–3.2	0.5 ± 1.3		
*Pila occidentalis*	0–11.8	3.7 ± 5	0–3.5	0.5 ± 1.4	0–5.7	1.2 ± 2.2	0–6.5	1.8 ± 2.5
*Gyrinus* sp.	0–10.5	2.2 ± 4.1	0–7.1	2.3 ± 3.3	0–3.8	1.4 ± 1.6	0–8.5	1.9 ± 3.4
*Haliplus* sp.	0–2.6	0.8 ± 1.3	0–11.2	3.4 ± 5	0–5.8	1.7 ± 2.6	0–26.1	4.9 ± 10.5
*Crenigomphus* sp.					0–3.2	0.9 ± 1.4	0–5	1 ± 2
*Dixella* sp.	0–5.3	1.1 ± 2.1	0–10	4.5 ± 4.3	0–2.9	0.5 ± 1.1	0–9.8	3.2 ± 4
Oligoneuriidae			0–1.4	0.6 ± 0.6	0–11.4	2.8 ± 4.7	0–1	0.1 ± 0.4
*Pinheyschna* sp.	0–1	0.3 ± 0.5			0–5.7	1.6 ± 2.1	0–7.1	1.1 ± 2.9
*Ephemerellina* sp.	0–2.7	0.7 ± 1.2	0–2.8	1 ± 1.1			0–11.4	2.4 ± 4.5

Aquatic insects and molluscs accounted for a larger share of relative abundance at 1150–1199 m and above 1200 m than below 1100 m (Table 2). At 1150–1199 m, contributions were spread across Coleoptera (e.g., *Rhantus* sp., 0.8% ± 1.3%; *Gyrinus* sp., 1.4% ± 1.6%), Hemiptera (*Laccocoris* sp., 5.6% ± 9.1%; Nepidae, 2.8% ± 3.1%), Odonata (*Anax* sp., 2.6% ± 4.5%) and Gastropoda (1.7% ± 1.9%), with no single insect or mollusc taxon exceeding 6% (Table 2). Above 1200 m, mean total abundance was the lowest recorded (60.6 ± 23.4 individuals per pan, against 232.8 ± 236.9 at 1100–1149 m; Table S1), and the most abundant taxa after the branchiopods were *Haliplus* sp. (4.9% ± 10.5%), *Gyraulus connollyi* (3.0% ± 3.5%), *Hydrocanthus* sp. (3.0% ± 3.7%) and *Gerris swakopensis* (2.9% ± 2.6%; Table 2).

Global ANOSIM detected no separation of assemblages among elevation classes (Global *R* = −0.004, *p* = 0.571), and all pairwise *R* values were low (*R* < 0.18), including those for the contrasts that were significant under PERMANOVA. The two tests together indicate that among‐class differences in composition are small relative to within‐class variability, and that assemblages vary continuously across the aquifer rather than falling into discrete elevational groups.

The SIMPER analysis further demonstrated moderate within‐group similarity (26.1%–34.7%) and high between‐group dissimilarity (67.9%–73.4%), driven primarily by a limited number of dominant taxa (Table 3). Across all elevations, Anostraca consistently accounted for a large proportion of within‐group similarity (43%–55%), highlighting its ecological dominance (Table 3). Low and mid‐elevation communities were additionally characterised by taxa such as Notostraca, Spinicaudata, *Pila occidentalis*, and *Rhantus* sp., whereas high‐elevation assemblages included distinct contributors such as *Gerris swakopensis* , *Gyraulus connollyi*, *Dixella* sp., and *Hydrocanthus* sp. (Table 3). Between‐group dissimilarity was largely driven by shifts in the relative abundance or presence of dominant taxa, with Anostraca contributing 16%–18% of dissimilarity across comparisons (Table 3). Spinicaudata and Notostraca further contributed 5%–8% and 5%–7%, respectively, with the absence of Spinicaudata at high elevations amplifying compositional turnover (Table 3). Elevation‐specific patterns revealed strong dominance of large branchiopods at low elevations, gradual compositional shifts at mid‐elevations, and more distinct assemblages at high elevations characterised by the emergence of additional insect and mollusc taxa and the loss of some lower‐elevation specialists (Table 3).

**TABLE 3:** SIMPER analysis of macroinvertebrate communities across elevation categories. Values are average within‐group similarities and average between‐group dissimilarities (%), followed by the taxa contributing most to each.

Comparison	Average similarity or dissimilarity (%)	Top contributing taxa (%)
*Within‐group similarities*		
< 1100 m	34.65	Anostraca (51.56), Notostraca (14.66), Spinicaudata (12.39), *Pila occidentalis* (1.66), *Rhantus* sp. (1.51)
1100–1149 m	29.55	Anostraca (43.82), Spinicaudata (20.19), Notostraca (7.73), *Pila occidentalis* (3.24), *Hydrocanthus* sp. (2.75), *Rhantus* sp. (2.01)
1150–1199 m	30.11	Anostraca (50.34), Spinicaudata (11.11), Notostraca (9.24), *Rhantus* sp. (1.93), *Pila occidentalis* (1.24)
> 1200 m	26.07	Anostraca (54.06), Notostraca (14.72), *Gerris swakopensis* (4.99), *Gyraulus connollyi* (3.63), *Dixella* sp. (3.55), *Hydrocanthus* sp. (3.29), *Sigara* sp. (1.78)
*Between‐group dissimilarities*		
< 1100 m and 1100–1149 m	68.52	Anostraca (17.65), Spinicaudata (8.2), Notostraca (6.91), *Pila occidentalis* (4.71), *Hydrocanthus* sp. (3.34), *Rhantus* sp. (3.08), *Sigara* sp. (1.83)
< 1100 m and 1150–1199 m	67.94	Anostraca (17.8), Spinicaudata (7.2), Notostraca (7.02), *Rhantus* sp. (2.96), *Pila occidentalis* (2.92), *Hydrocanthus* sp. (2.14), *Anisops* sp. (1.73)
1100–1149 m and 1150–1199 m	70.65	Anostraca (16.97), Spinicaudata (8.44), Notostraca (5.36), *Pila occidentalis* (4.32), *Rhantus* sp. (3.31), *Hydrocanthus* sp. (3.0), *Haliplus* sp. (1.67)
< 1100 m and > 1200 m	70.24	Anostraca (16.62), Notostraca (6.82), Spinicaudata (6.32), *Haliplus* sp. (3.81), *Rhantus* sp. (3.27), *Pila occidentalis* (2.49), *Hydrocanthus* sp. (2.41), *Dixella* sp. (2.38)
1100–1149 m and > 1200 m	73.38	Anostraca (15.68), Spinicaudata (8.41), Notostraca (5.84), *Haliplus* sp. (3.93), *Pila occidentalis* (3.87), *Rhantus* sp. (3.55), *Hydrocanthus* sp. (2.89)
1150–1199 m and > 1200 m	71.97	Anostraca (16.16), Notostraca (5.99), Spinicaudata (5.91), *Haliplus* sp. (3.79), *Rhantus* sp. (3.52), *Dixella* sp. (2.39), *Gyraulus connollyi* (2.29), *Hydrocanthus* sp. (2.23), *Pila occidentalis* (2.07)

#### 3.2.1 Macroinvertebrate Metrics

Mean taxa richness ranged from 15.1 ± 1.4 taxa above 1200 m to 20.1 ± 1.1 taxa at 1100–1149 m (Table S1) and differed significantly among classes (*F* = 5.045, *p* = 0.002; Table 1). Tukey tests located this difference between 1100–1149 and 1150–1199 m (*p* = 0.020) and between 1150–1199 and > 1200 m (*p* = 0.045); no other contrast was significant (Table S2). Mean abundance was highest at 1100–1149 m (232.8 ± 236.9 individuals) and lowest above 1200 m (60.6 ± 23.4), but did not differ significantly among classes (*F* = 1.007, *p* = 0.390). Simpson's index (class means 0.7–0.8), Shannon–Wiener diversity (2.1–2.3) and Pielou's evenness (0.5–0.6) were similar across classes and showed no significant differences (*F* = 0.195, *p* = 0.900; *F* = 0.829, *p* = 0.478; *F* = 0.661, *p* = 0.577, respectively; Table 1). Within‐class variation exceeded the differences among class means for every metric; richness, for example, spanned 16–24 taxa within the 1150–1199 m class alone (Table S1).

#### 3.2.2 Relationship Between Macroinvertebrates, Elevation and Environmental Variables

Taxa richness, Shannon–Wiener diversity, Simpson's diversity, and Pielou's evenness were not significantly correlated with altitude (*r* = 0.09, *p* = 0.063; *r* = 0.09, *p* = 0.063; *r* = 0.05, *p* = 0.255; *r* = 0.07, *p* = 0.159, respectively). Latitude was weakly but significantly correlated with Shannon–Wiener diversity (*r* = 0.11, *p* = 0.024) and evenness (*r* = 0.10, *p* = 0.034), but not with taxa richness (*r* = 0.05, *p* = 0.304) or Simpson's diversity (*r* = 0.09, *p* = 0.056). Longitude was weakly correlated with taxa richness (*r* = 0.15, *p* = 0.002), but not with Shannon–Wiener diversity (*r* = 0.09, *p* = 0.056), Simpson's diversity (*r* = 0.04, *p* = 0.369), or evenness (*r* = 0.05, *p* = 0.299) (Figure 2). All significant correlations were weak, accounting for less than 3% of the variance in the metric concerned, and *p*‐values were not adjusted for multiple comparisons.

**FIGURE 2:**
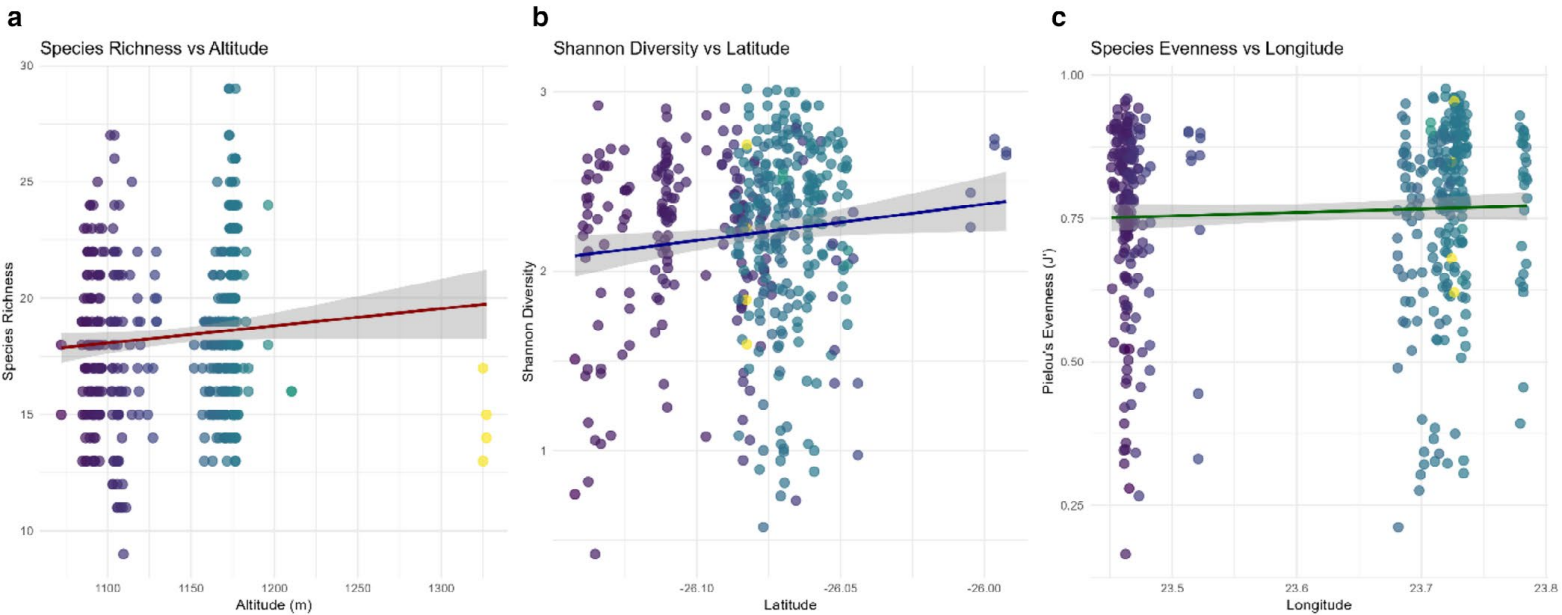
Relationships between macroinvertebrate diversity metrics and spatial position across the 221 surveyed pans: (a) taxa richness against altitude, (b) Shannon–Wiener diversity against latitude and (c) Pielou's evenness against longitude. Solid lines show linear fits with 95% confidence bands. All relationships are weak (*r* < 0.15), and within‐class variation exceeds the differences among elevation classes.

The predictive performance of boosted regression trees (BRT) models relating macroinvertebrate taxa richness to environment variables was as follows: 4000 number of trees fitted (boosted models), with a mean total and residual deviance of 0.589 and 0.001, respectively. The estimated cross–validated deviance ± standard error was 0.001 ± 0.001 and the cross–validated correlation of 0.99 (with a training data correlation of 0.98). 99.9% of variation was explained. In the boosted regression tree model of taxa richness, geographic position accounted for almost all of the relative influence: longitude 76.8%, altitude 14.5% and latitude 8.6%, with every measured physicochemical variable contributing < 0.04% (pH 0.04%, dissolved oxygen 0.04%, conductivity 0.01%; Figure 3). The fitted functions for longitude, altitude and latitude were step‐like rather than gradual (Figure 3), indicating that the model partitions pans into a small number of spatial groups rather than describing continuous responses to underlying gradients. Model parameterisation and performance under both random and spatially blocked cross‐validation are reported in the Methods; the contrast between them indicates the degree to which apparent accuracy derives from spatial autocorrelation among neighbouring pans. The model is therefore reported as a ranking of the relative influence of predictors within this survey, and no predictive interpretation is drawn from it.

**FIGURE 3:**
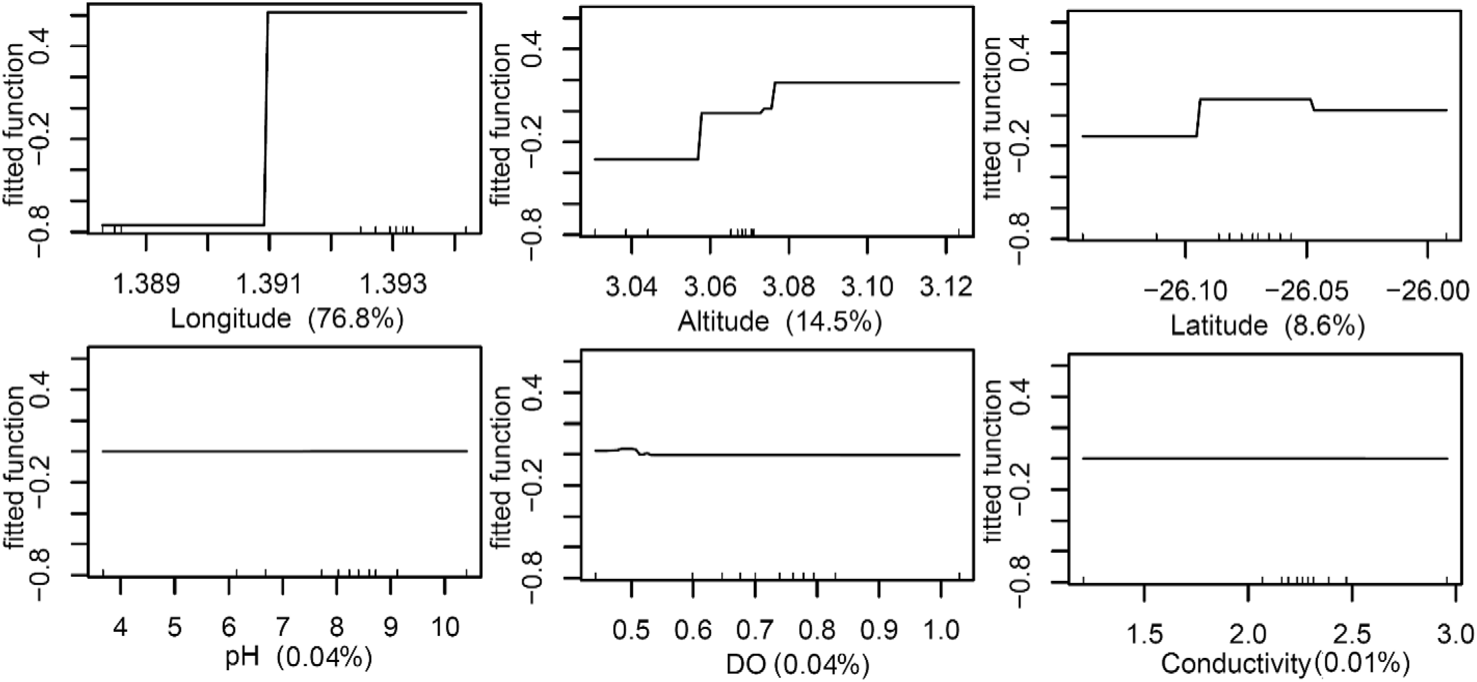
Fitted functions (partial dependence) for the six most influential predictors in the boosted regression tree model of macroinvertebrate taxa richness. Relative influence (%) is given in parentheses after each predictor name, and predictor values are shown on the scale used for model fitting.

## 4 Discussion

This study examined how local environmental conditions and landscape position relate to macroinvertebrate assemblages in the groundwater‐dependent pan wetlands of the KBTA. Three results frame the interpretation that follows. First, assemblages differed statistically among pans grouped by elevation, but the effect was weak and the associated environmental differences lay in hydroperiod‐related variables, water depth, dissolved oxygen, temperature and nutrients, rather than in elevation itself; across the approximately 200 m of relief surveyed here, elevation cannot plausibly act as a climatic filter and is best read as a surrogate for topographic and hydrogeomorphic setting. Second, compositional change was expressed as shifts in the relative abundance of a few dominant taxa rather than as species turnover, so richness‐ and diversity‐based metrics were largely insensitive to it. Third, geographic position, and longitude in particular, accounted for more variation in taxa richness than any measured water‐chemistry variable, which we interpret as evidence of spatially structured habitat variation that was not measured directly.

Physicochemical conditions across the pans reflected a strong groundwater signature, with predominantly neutral to alkaline pH consistent with the carbonate‐rich geology of the aquifer (Gomo and Ngobe 2024). The wide pH and oxidation–reduction potential ranges recorded in the lower‐lying pans are consistent with episodic surface runoff inputs and evaporative concentration in shallow systems with fluctuating hydroperiods, whereas the few pans above 1200 m were consistently alkaline, oxygen‐poor and periodically reducing, as expected of deeper water bodies with longer residence times and reduced mixing (Masina et al. 2023). These contrasts track depth and hydroperiod rather than elevation, and are consistent with wider evidence that groundwater–surface water exchange governs the chemical setting of pan systems in this aquifer (Rampheri et al. 2023; Gomo and Ngobe 2024).

Water depth, and by extension hydroperiod, is the most plausible proximate control on the assemblages recorded here. Depth differed significantly among pan classes and was not monotonically related to elevation, being lowest at 1150–1199 m and greatest in the small number of pans above 1200 m; temperature and nutrients showed the same non‐monotonic pattern. Deeper pans hold water for longer, buffer thermal extremes, and allow taxa with protracted development to complete their life cycles, whereas shallow pans dry rapidly and select for desiccation‐tolerant strategies (Batzer and Boix 2016; Shrestha et al. 2021). Nutrient concentrations likewise varied by class rather than along a gradient, with ammonium highest in the shallowest and most frequently drying pans and phosphate highest where redox conditions were most reducing, a pattern consistent with internal loading and suppressed nitrification under low oxygen (Taylor et al. 2021). Enrichment of this kind can raise productivity and invertebrate abundance, but sustained enrichment favours tolerant taxa and erodes compositional distinctiveness (Wagenhoff et al. 2012; Wurtsbaugh et al. 2019).

Compositional change across the pan network was driven by the relative dominance of large branchiopods. Anostraca, Spinicaudata and Notostraca were most abundant in the shallow pans, where rapid development and desiccation‐resistant resting eggs allow populations to complete a generation before drying (Van den Broeck et al. 2015; Batzer and Boix 2016). Their decline in deeper pans is unlikely to reflect intolerance of stable conditions. Large branchiopods are poorly defended, conspicuous swimmers whose persistence depends largely on the absence of predators; longer hydroperiods allow predatory Coleoptera, Hemiptera and Odonata to colonise and establish, and these predators suppress branchiopod populations (Batzer and Boix 2016; Gerstle et al. 2023). The reciprocal pattern recorded here, declining branchiopod dominance alongside increasing representation of predatory insects and of molluscs, is therefore better explained by the joint action of hydroperiod and predation than by habitat stability alone. The same mechanism explains why richness and diversity metrics changed so little across classes: the taxa that increase largely replace branchiopod individuals rather than adding to an otherwise unchanged assemblage.

The pans above 1200 m were deeper, warmer, more alkaline and markedly oxygen‐poor (2.4 ± 0.4 mg L^−1^), and supported the lowest abundance and richness but the highest evenness. Such conditions favour hypoxia‐tolerant taxa and exclude oxygen‐sensitive groups (Harrison et al. 2018; Obertegger et al. 2021), and they are consistent with reports that long‐hydroperiod, low‐oxygen wetlands support specialised rather than diverse assemblages (Schriever et al. 2014). This class contained only three pans, however, and we therefore treat it as indicative rather than as a characterisation of high‐lying pans within the aquifer. The disproportionate leverage that these three sites exert on among‐class tests and on the boosted regression tree models is a feature of the survey design rather than an ecological result.

Importantly, diversity indices such as Shannon and Simpson remained relatively stable across elevation gradients, indicating that changes in community structure were driven primarily by shifts in dominance and relative abundance rather than species loss. This pattern suggests a degree of functional redundancy within macroinvertebrate communities, which may confer resilience to moderate environmental fluctuations but can obscure early ecological degradation if assessments rely solely on richness–based metrics (Lechêne et al. 2018). The persistence of key taxa such as Anostraca across all elevation bands further highlights the buffering role of groundwater connectivity, which supports both transient and more stable assemblages across the landscape (Yihdego et al. 2017; Deane et al. 2018).

Spatial position accounted for more variation in taxa richness than any measured local variable, but that result should not be overstated. The boosted regression tree model assigned virtually all relative influence to longitude, altitude and latitude combined, and the corresponding fitted functions were step functions rather than gradual responses. This is the signature expected when a model is fitted to sites that are strongly clustered in space: coordinates act as identifiers of sampling clusters, and randomly drawn cross‐validation folds contain near neighbours of the sites they are used to predict, which inflates apparent accuracy (Roberts et al. 2017). We therefore read the spatial signal as evidence that pans differ systematically among clusters in ways not captured by single‐visit measurements of water chemistry, plausibly in hydroperiod, catchment area, depth to groundwater, livestock use and proximity to neighbouring pans, rather than as evidence that geographic coordinates predict richness. Distance decay of assemblage similarity was not tested directly in this study, so dispersal limitation and spatially structured environmental variation cannot be separated with these data; metacommunity studies of pond and pan networks nevertheless consistently find that both contribute alongside local chemistry (Heino et al. 2017; Sarremejane et al. 2022; Guerrero et al. 2025).

The weakness of the altitudinal and latitudinal signals follows from the geometry of the study area: approximately 200 m of relief and less than 0.15° of latitude leave little scope for climatic differentiation, and studies that detect elevation effects on aquatic assemblages typically span ranges an order of magnitude larger (Milner et al. 2023; Hou et al. 2025). The longitudinal contrast was the strongest spatial signal, with richness slightly higher in the western than in the eastern pan clusters (Figure 1). Of the possible explanations, differences in pan density and hydroperiod between the western and eastern clusters are the most likely, since closely spaced pans shorten inter‐pan dispersal distances and widen the range of hydroperiods available within dispersal range. Differences in water quality are the least likely, because no measured physicochemical variable retained appreciable influence in the models. This interpretation is testable and should be addressed by explicitly measuring inter‐pan distance, pan density and hydroperiod. Consistent with it, SIMPER attributed among‐class differences to changes in the dominance of a few taxa rather than to species replacement, indicating a continuum of assemblage composition across the aquifer rather than discrete ecological zones (Arts et al. 2022; Ramírez 2024).

From a conservation perspective, these findings underscore the vulnerability of groundwater‐dependent pan wetlands to hydrological alteration, land‐use change and climate variability. Because assemblage structure tracked depth, oxygen and nutrient status rather than elevation, the practical priority is maintenance of the groundwater regime and of the full range of hydroperiods represented across the pan network (Chiloane et al. 2022; Rampheri et al. 2023). Our data do not identify any single elevation class as a biodiversity hotspot: richness and diversity varied more within than among classes, so protecting a representative set of pans spanning the observed range of depths, hydroperiods and cluster positions is more defensible than targeting one elevation band. The prominence of spatially structured variation likewise argues for landscape‐scale rather than site‐scale protection, retaining clusters of pans together with the matrix between them (Morris et al. 2019; Graziano et al. 2022). Effective management of the KBTA requires coordinated transboundary governance to regulate abstraction and to limit cumulative impacts (Loucks and Van Beek 2017), and monitoring should track assemblage composition and relative abundance rather than richness alone, which was insensitive to the compositional changes recorded here (Lechêne et al. 2018; Mureithi et al. 2025).

The limitations of the study were that each pan was visited once, so hydroperiod was inferred from depth and morphometry rather than measured, and seasonal turnover in these systems is likely to be substantial. Elevation classes were unequally represented (*n* = 55, 40, 123 and 6), which constrains among‐class inference and gives the three highest pans disproportionate leverage. Furthermore, macroinvertebrates were identified to the lowest practicable level, which was genus for some groups and family or subfamily for others, so richness values are not directly comparable with studies applying uniform taxonomic resolution. Distance decay of assemblage similarity was not tested, and the boosted regression tree models are correlative and were fitted to spatially clustered data. Multi‐season sampling, direct measurement of hydroperiod, pan density and inter‐pan distance, and trait‐based analysis are needed to test the mechanisms proposed here.

## 5 Conclusion

Macroinvertebrate assemblages in the pan wetlands of the Khakhea–Bray Transboundary Aquifer are structured by hydroperiod‐related conditions such as water depth, oxygen availability, and nutrient status, and by spatially structured variation among pan clusters, rather than by elevation, which across the modest relief of this landscape functions only as a surrogate for topographic setting. Compositional change was expressed through shifts in the dominance of a few taxa, principally large branchiopods, rather than through species loss, so richness‐based metrics alone will not detect ecological change in these systems. Safeguarding this fauna therefore depends on maintaining groundwater regimes and the full range of hydroperiods across the pan network, and on transboundary coordination of abstraction, rather than on the protection of individual sites.

## Author Contributions

**Busisiwe P. Gumede:** data curation (equal), formal analysis (equal), investigation (equal), validation (equal), visualization (equal), writing – original draft (equal), writing – review and editing (equal). **Linton F. Munyai:** funding acquisition (equal), investigation (equal), supervision (equal), validation (equal), visualization (equal), writing – original draft (equal), writing – review and editing (equal). **Naicheng Wu:** funding acquisition (equal), methodology (equal), validation (equal), visualization (equal), writing – original draft (equal), writing – review and editing (equal). **Collins Oduro:** formal analysis (equal), methodology (equal), validation (equal), visualization (equal), writing – original draft (equal), writing – review and editing (equal). **Tatenda Dalu:** conceptualization (lead), data curation (equal), formal analysis (lead), funding acquisition (lead), investigation (lead), methodology (lead), project administration (lead), resources (lead), software (equal), supervision (lead), validation (equal), visualization (equal), writing – original draft (equal), writing – review and editing (equal).

## Funding

The study was funded by the National Research Foundation Thuthuka grant (UID: TTK240409213108; TTK230508103682). NWU was funded by the National Natural Science Foundation of China (no. 52279068). LFM acknowledges funding under the Junior Research Fellows Programme of the Department of Higher Education (DHET) University Capacity Development Programme (UCDP).

## Ethics Statement

The permits were granted by the North West Department of Economic Development, Environment, Conservation and Tourism (permit number: NW58642/01/2025). The study has been ethically approved by University of Mpumalanga Animal Sciences Research Ethics Committee number: UMP/DALU/FANS/2025/2 and University of Mpumalanga School of Biology and Environmental Sciences Research Ethics Committee ethics number: AS/UMP/Gumede201728788/BIO/MSc/2025/1.

## Consent

All authors agreed to participate in this manuscript and made meaningful contributions.

## Conflicts of Interest

The authors declare no conflicts of interest or personal relationships that could have appeared to influence the work reported in this paper. Prof Tatenda Dalu is an Associate Editor of the journal.

## Supporting information


**Table S1:** Environmental variables and macroinvertebrate metrices recorded among pan wetlands at different elevations within the Khakhea–Bray Transboundary aquifer region, South Africa. DO, dissolved oxygen; ORP, oxygen reduction potential; TDS, total dissolved solids.
**Table S2:** Tukey's posthoc analysis of environmental variables and macroinvertebrate metricises recorded among pan wetlands at different elevations within the Khakhea–Bray Transboundary aquifer region, South Africa.

## Data Availability

The species‐by‐site matrix, the environmental data and the R scripts used for all analyses reported here have been submitted with this manuscript for review.
