# Supplementary material for: Spatial and Environmental Drivers of Macroinvertebrate Diversity in Groundwater‐Dependent Pan Wetlands Across a Transboundary Aquifer System
Source: Ecol Evol. 2026 Oct 19;16(10):e74440. doi: 10.1002/ece3.74440 (PMC13680134; doi:10.1002/ece3.74440)
Supplement: Supplementary file 1 [file ECE3-16-e74440-s001.docx]

# **Supplementary files**

**Table S1.** Environmental variables and macroinvertebrate metrices recorded among pan wetlands at different elevations within the Khakhea–bray Transboundary aquifer region, South Africa. Abbreviations: ORP – oxygen reduction potential, DO – dissolved oxygen, TDS – total dissolved solids,

| **Taxa** | **<1 100 m (*n* = 55)** | | **1 100–1 149 m (*n* = 40)** | | **1 150–1 199 m (*n* = 123)** | | **>1 200 m (*n* = 3)** | |
| --- | --- | --- | --- | --- | --- | --- | --- | --- |
|  | **Range** | **Mean ± SD** | **Range** | **Mean ± SD** | **Range** | **Mean ± SD** | **Range** | **Mean ± SD** |
| ***Environmental*** | | | | | | | | |
| pH | 3.6 – 10.1 | 7.7 ± 1.4 | 3.6 – 10.1 | 7.6 ± 1.4 | 4.8 – 10.4 | 7.9 ± 1.1 | 7.9 – 8.9 | 8.6 ± 0.4 |
| ORP (mV) | 194.1 – 2 009 | 513.7 ± 732.6 | 130.1 – 184.9 | 159.6 ± 19 | 22.8 – 226.8 | 97.7 ± 75.8 | –98.7 – 161 | 102.1 ± 99.7 |
| DO (mg/L) | 4.8 – 5.8 | 5.2 ± 0.3 | 4.8 – 5.3 | 5.1 ± 0.1 | 2.4 – 6.7 | 4.7 ± 1.8 | 2 – 3.1 | 2.4 ± 0.4 |
| Conductivity (μS/cm) | 164 – 342 | 242.3 ± 68.2 | 106 – 402 | 186.5 ± 109.6 | 192 – 445 | 301 ± 99.9 | 119 – 216 | 167.6 ± 37.4 |
| TDS (mg/L) | 96 – 221 | 133 ± 46.1 | 60 – 94 | 81 ± 15.8 | 131 – 221 | 157.8 ± 34.5 | 68 – 108 | 86.1 ± 16.1 |
| Temperature | 12.4 – 13.8 | 13 ± 0.5 | 11.5 – 19.7 | 17.2 ± 3.1 | 12.1 – 17.6 | 15 ± 2.5 | 16 – 19 | 17.7 ± 1.1 |
| Surface area (m^2^) | 50 – 1500 | 550 ± 736.2 | 50 – 30 000 | 10 033.3 ± 15 466.1 | 200 – 20 000 | 7 566.6 ± 9 685.5 | 150 – 5 500 | 2 883.3 ± 2 394.2 |
| Water depth (cm) | 4 – 10 | 7.7 ± 2.7 | 15 – 34 | 21.3 ± 6.9 | 5 – 15 | 8.3 ± 4 | 9.4 – 61 | 34.7 ± 26.6 |
| Ammonium (mg/L) | 0 – 63 | 21.1 ± 32.4 | 3.1 – 10.7 | 5.7 ± 3.8 | 0 – 4.6 | 1.6 ± 2.3 | 1.1 – 24.4 | 10.3 ± 11 |
| Nitrate (mg/L) | 0 – 77.6 | 34.3 ± 35.3 | 18.2 – 104.8 | 71.7 ± 41.8 | 11.6 – 24.4 | 16.2 ± 6.3 | 6.7 – 29.6 | 16.5 ± 10.5 |
| Phosphate (mg/L) | 0 – 5.1 | 1.7 ± 2.6 | 0 – 5.9 | 2.3 ± 2.8 | 4.1 – 8 | 6.5 ± 1.9 | 2.2 – 9.4 | 5.3 ± 3.3 |
| ***Macroinvertebrate metrices*** | | | | | | | | |
| Taxa richness | 16 – 23 | 19.6 ± 3 | 19 – 22 | 20.1 ± 1.1 | 16 – 24 | 19.3 ± 3 | 13 – 17 | 15.1 ± 1.4 |
| Abundance | 72 – 118 | 85 ± 17.7 | 70 – 595 | 232.8 ± 236.9 | 34 – 210 | 106.5 ± 74 | 35 – 91 | 60.6 ± 23.4 |
| Simpson | 0.6 – 0.9 | 0.8 ± 0 | 0.3 – 0.9 | 0.7 ± 0.2 | 0.7 – 0.9 | 0.8 ± 0 | 0.6 – 0.9 | 0.8 ± 0.1 |
| Shannon–Wiener | 1.8 – 2.7 | 2.3 ± 0.3 | 0.9 – 2.7 | 2.1 ± 0.7 | 2 – 2.7 | 2.3 ± 0.2 | 1.5 – 2.7 | 2.2 ± 0.4 |
| Evenness | 0.3 – 0.7 | 0.5 ± 0.1 | 0.1 – 0.7 | 0.5 ± 0.2 | 0.3 – 0.8 | 0.5 ± 0.2 | 0.3 – 0.8 | 0.6 ± 0.2 |
| Altitude | 1 096.5 – 1 098.1 | 1 097.1 ± 0.7 | 1 128.3 – 1 129.4 | 1 129 ± 0.5 | 1 182.9 – 1 196.1 | 1 187.8 ± 6.4 | 1 210.2 – 1 327 | 1 287.4 ± 59.7 |

**Table S2.** Tukey’s posthoc analysis of environmental variables and macroinvertebrate metricises recorded among pan wetlands at different elevations within the Khakhea–bray Transboundary aquifer region, South Africa.

| **Dependent variable** | **Altitude 1** | **Altitude 2** | ***p*** | **Dependent variable** | **Altitude 1** | **Altitude 2** | ***p*** |
| --- | --- | --- | --- | --- | --- | --- | --- |
| Dissolved oxygen | <1100 | 1100–1149 | 0.683 | Nitrate | <1100 | 1100–1149 | **<0.001** |
|  | <1100 | 1150–1199 | 0.054 |  | <1100 | 1150–1199 | 0.786 |
|  | <1100 | >1200 | **<0.001** |  | <1100 | >1200 | 0.955 |
|  | 1100–1149 | 1150–1199 | **0.003** |  | 1100–1149 | 1150–1199 | **<0.001** |
|  | 1100–1149 | >1200 | **<0.001** |  | 1100–1149 | >1200 | 0.251 |
|  | 1150–1199 | >1200 | **<0.001** |  | 1150–1199 | >1200 | 0.994 |
| Temperature | <1100 | 1100–1149 | 1.000 | Phosphate | <1100 | 1100–1149 | **<0.001** |
|  | <1100 | 1150–1199 | **<0.001** |  | <1100 | 1150–1199 | 0.624 |
|  | 1100–1149 | 1150–1199 | **<0.001** |  | 1100–1149 | 1150–1199 | **<0.001** |
|  | 1150–1199 | >1200 | 0.924 |  | 1150–1199 | >1200 | 0.978 |
|  | >1200 | <1100 | 0.627 |  | >1200 | <1100 | 1.000 |
|  | >1200 | 1100–1149 | 0.634 |  | >1200 | 1100–1149 | 0.220 |
| Water depth | <1100 | 1100–1149 | **<0.001** | Taxa richness | <1100 | 1100–1149 | 0.697 |
|  | <1100 | 1150–1199 | 0.472 |  | <1100 | 1150–1199 | 0.244 |
|  | <1100 | >1200 | 0.112 |  | <1100 | >1200 | 0.164 |
|  | 1100–1149 | 1150–1199 | **<0.001** |  | 1100–1149 | 1150–1199 | **0.020** |
|  | >1200 | 1100–1149 | 1.000 |  | >1200 | 1100–1149 | 0.338 |
|  | >1200 | 1150–1199 | 0.239 |  | >1200 | 1150–1199 | **0.045** |
| Ammonium | <1100 | 1100–1149 | 0.466 |  |  |  |  |
|  | <1100 | 1150–1199 | **<0.001** |  |  |  |  |
|  | <1100 | >1200 | 0.708 |  |  |  |  |
|  | 1100–1149 | 1150–1199 | **0.010** |  |  |  |  |
|  | 1150–1199 | >1200 | 0.978 |  |  |  |  |
|  | >1200 | 1100–1149 | 0.945 |  |  |  |  |
